# Key Regulators of Autophagosome Closure

**DOI:** 10.3390/cells10112814

**Published:** 2021-10-20

**Authors:** Wenyan Jiang, Xuechai Chen, Cuicui Ji, Wenting Zhang, Jianing Song, Jie Li, Juan Wang

**Affiliations:** 1 Center of Excellence for Environmental Safety and Biological Effects, Faculty of Environment and Life Sciences, Beijing International Science and Technology Cooperation Base for Antiviral Drugs, Beijing University of Technology, 100 Ping Le Yuan, Chaoyang District, Beijing 100124, China; 18865386696@163.com (W.J.); chenxuechai@bjut.edu.cn (X.C.); hiiamjcc@163.com (C.J.); 17600398559@163.com (W.Z.); 15650272551@163.com (J.S.); 2 Laboratory for Marine Fisheries and Aquaculture, Qingdao National Laboratory for Marine Science and Technology, Key Laboratory of Maricultural Organism Disease Control, Ministry of Agriculture and Rural Affairs, Yellow Sea Fisheries Research Institute, Chinese Academy of Fishery Sciences, Qingdao 266071, China; lijie@ysfri.ac.cn

**Keywords:** autophagy, autophagosome, closure, isolation membrane

## Abstract

Autophagy is an evolutionarily conserved pathway, in which cytoplasmic components are sequestered within double-membrane vesicles called autophagosomes and then transported into lysosomes or vacuoles for degradation. Over 40 conserved autophagy-related (ATG) genes define the core machinery for the five processes of autophagy: initiation, nucleation, elongation, closure, and fusion. In this review, we focus on one of the least well-characterized events in autophagy, namely the closure of the isolation membrane/phagophore to form the sealed autophagosome. This process is tightly regulated by ESCRT machinery, ATG proteins, Rab GTPase and Rab-related proteins, SNAREs, sphingomyelin, and calcium. We summarize recent progress in the regulation of autophagosome closure and discuss the key questions remaining to be addressed.

## 1. Introduction

**Figure 1:**
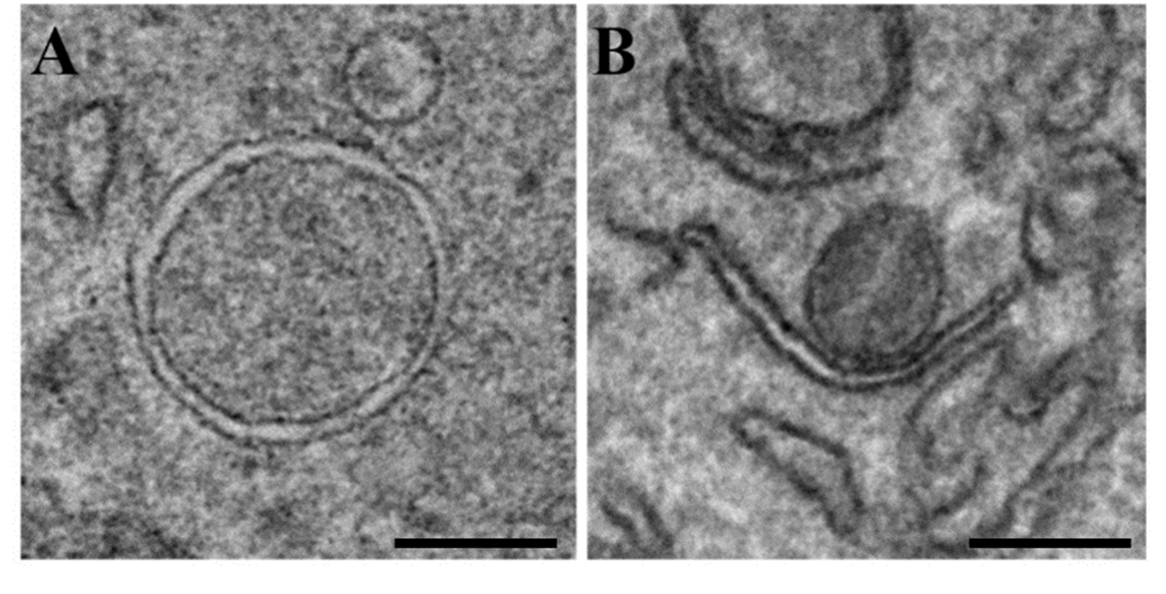
Transmission electron microscopic images of autophagosome (**A**) and isolation membrane (**B**). Neuro-2a (N2a) cells were starved in EBSS for 2 h. Scale bars, 200 nm.

Autophagy is a highly conserved pathway, in which cytoplasmic components are sequestered within double-membrane vesicles called autophagosomes, and transported into lysosomes or vacuoles for degradation [1,2,3]. Autophagy is essential for maintaining energy homeostasis and protecting cells against stress and plays important roles during development and disease. Numerous human pathologies, including infectious diseases, neurodegenerative disorders, and cancer have been associated with defects in the autophagy pathway [4,5,6]. Starvation-induced macroautophagy is non-selective; in contrast, specific cargo receptors are required for recognition of cellular cargoes during selective autophagy [1,2,3]. Autophagy is initiated with the de novo formation of a crescent-shaped membrane, known as the isolation membrane (IM) or phagophore (Figure 1). IMs sequester cytoplasmic cargo, expand and seal to form fully closed autophagosomes [1,2,3,4,5,6]. In mammalian cells, autophagosomes then fuse with endolysosomal compartments to form degradative autolysosomes and facilitate degradation of the sequestered material. Autophagy is regulated by autophagy-related (ATG) proteins, which are recruited to the site of autophagosome formation in a hierarchical order upon autophagy induction [1,2,3,4,5,6]. Autophagy-related proteins include the ULK1/Atg1 complex, required for the initiation of autophagy, the PI3K complex, responsible for PI3P production and essential for nucleation of the IM, Atg9, the only transmembrane core ATG protein required during the early stages of autophagy and interacting with lipid transfer protein Atg2 and PI3P-effector Atg18 (WIPIs), and two ubiquitin-like conjugation systems: the Atg8 conjugation to phosphatidylethanolamine (PE), the conjugation of Atg12–Atg5, co-localized with Atg16 in the IM. In the Atg12–Atg5 conjugation system, Atg7 and Atg10 function as E1- and E2-like enzymes, respectively. In the Atg8 conjugation system, Atg7 functions as an E1-like enzyme, and Atg3 acts as an E2-like enzyme. The Atg12–Atg5–Atg16 complex acts as an E3-like enzyme for the conjugation of Atg8s to PE. Atg8 is cleaved by Atg4 to expose a glycine residue before conjugation, and Atg8–PE is also cleaved by Atg4, which allows the recycling of Atg8 [1,2,3,4,5,6]. More than 40 conserved autophagy-related (*ATG*) genes define the core machinery for the five steps of autophagy: initiation, nucleation, elongation, closure, and fusion [1,7].

Among the least well-characterized events in autophagy is the closure of the IM to form sealed autophagosome, which was originally thought to be a fusion process. Recent research indicates that autophagosome closure does not necessarily require fusion, but involves fission of the inner and outer membrane of the IM [8]. Recent progress in the field has shown that the closure of autophagosomes is tightly regulated by ESCRT complexes, ATG proteins, SNAREs, Rab GTPase, Rab-related proteins, sphingomyelin, and calcium. In the following sections, we have summarized the current understanding of the key regulators of autophagosome closure.

## 2. Endosomal Sorting Complex Required for Transport (ESCRT) Machinery

The process of autophagosome closure is topologically related to the biogenesis of multivesicular bodies, virus budding, and cytokinesis, all of which are cellular membrane scission processes involving the endosomal sorting complex required for transport (ESCRT) machinery [9,10]. ESCRT proteins were first identified as a subgroup of VPS (vacuolar protein sorting) genes in yeast and then found to mediate various topologically related membrane scission events [9,10]. ESCRT proteins can be classified into ESCRT-0, ESCRT-I, ESCRT-II, ESCRT-III, and ESCRT-associated proteins [9,10]. During the biogenesis of multivesicular bodies, ESCRT-0, ESCRT-I, and ESCRT-II contain ubiquitin-binding subunits, mediate the sorting of ubiquitylated cargo, and recruit ESCRT-III to the scission site. ESCRT-III-interacting protein ALIX also binds to ubiquitin and recruits ESCRT-III. ESCRT-III is the principal machinery for the membrane abscission reaction, consisting of the charged multivesicular body (CHMP) proteins 1–7 and increased sodium tolerance-1 (IST1). ESCRT-III cooperates with the ATPase VPS4 to drive membrane scission. VPS4 also mediates the disassembly and recycling of ESCRT-III [9,10].

**Figure 2:**
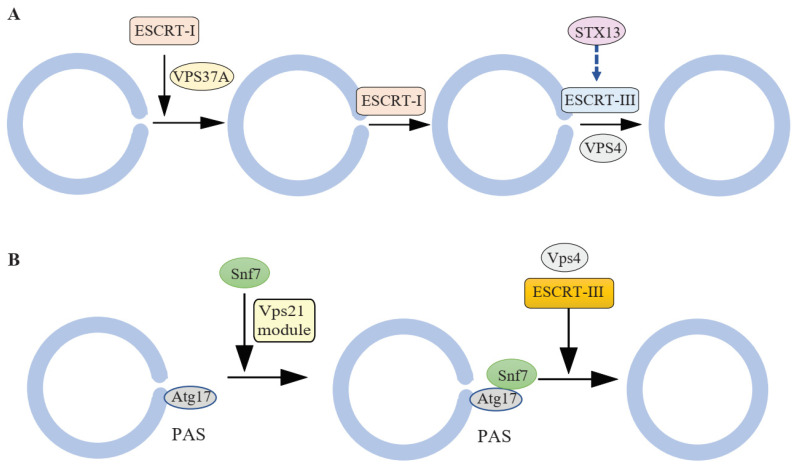
Model for ESCRT-mediated autophagosome closure. (**A**) In mammalian cells, the ESCRT-I complex translocates to the isolation membrane in a VPS37A-dependent manner, followed by recruitment of ESCRT-III. ESCRT-III and the AAA-ATPase VPS4 facilitate autophagosome closure. Syntaxin13 is a genetic modifier of the ESCRT-III component CHMP2B and functions upstream of ESCRT-III in autophagosome closure, although there is no direct physical interaction between Syntaxin13 and CHMP2B. (**B**) In budding yeast, the ESCRT-III subunit Snf7 interacts with the scaffold protein Atg17 in a Vps21-dependent manner, which results in the recruitment of ESCRT-III. ESCRT-III and Vps4 catalyze the closure of the isolation membrane to form a sealed autophagosome.

A possible role for the ESCRTs in autophagy was suggested more than a decade ago, as autophagic membrane structures were found to accumulate in ESCRT-depleted cells [11,12,13,14,15]. However, the nature of these autophagic abnormal membrane structures remained poorly understood, and the involvement of ESCRTs in the autophagy pathway was considered controversial. Several recent studies proposed a role for the ESCRT machinery in the closure of the IM to form a sealed autophagosome (Figure 2, Table 1). In 2018, Takahashi et al. developed a HaloTag-LC3-based autophagosome completion assay and revealed a role of the ESCRT-III subunit CHMP2A and the AAA-ATPase VPS4 in autophagosome closure [16]. CHMP2A translocates to the IM during autophagy, and its depletion leads to the accumulation of unclosed IMs. VPS4 works together with CHMP2A at the IM closure site for membrane fission [16]. The ESCRT-I subunit VPS37A was identified as a regulator of autophagosome closure from a HaloTag-LC3-based genome-wide screening. ESCRT-I complex translocates to the isolation membrane in a VPS37A dependent manner, followed by recruitment of ESCRT-III subunit CHMP2A [17]. The role of VPS37A or ESCRT-I in IM closure is independent of their function in the biogenesis of multivesicular bodies, as an N-terminal putative ubiquitin E2 variant domain in VPS37A is required for autophagosome closure, but is dispensable for multivesicular body formation. In addition, the endosome-specific ESCRT-I component UBAP1, is not required for autophagosome closure [17].

In the budding yeast *Saccharomyces cerevisiae*, the ESCRT-III subunit Snf7 (CHMP4 in mammals) and the Vps4 ATPase localize to the IM, and their depletion results in the accumulation of unsealed autophagosomes decorated with multiple Atg proteins [19,20]. Snf7 interacts with Atg17 (FIP200 in mammals) and leads to ESCRT recruitment to IM in a Vps21- (RAB5) dependent manner [19,20]. These studies indicate that ESCRT complexes play a role in autophagosome closure and shed light on the mechanisms by which ESCRTs are recruited to the closing IM.

The ESCRT machinery is similarly involved in autophagosome closure in selective autophagy. Zhen et al. reported that the ESCRT-III component CHMP4B is recruited to the unsealed autophagosome during macroautophagy and mitophagy [18]. They also found that CHMP2A mediates mitophagosome sealing, and the depletion of CHMP2A results in the accumulation of CHMP4B on mitophagosomes [18]. In budding yeast, the ESCRT-III subunit Snf7 and the ATPase Vps4 are involved in autophagosome closure during starvation-induced mitophagy. Snf7 interacts with Atg11, the scaffold protein in selective autophagy, which may be involved in the recruitment of ESCRT to IMs [35]. Interestingly, the interaction between Snf7 and Atg11 is not dependent on Vps21, suggesting the mechanism is different between selective and non-selective autophagy. All these studies indicate that ESCRT complexes play an essential role in autophagosome closure during both non-selective and selective autophagy.

## 3. ATG Proteins

**Figure 3:**
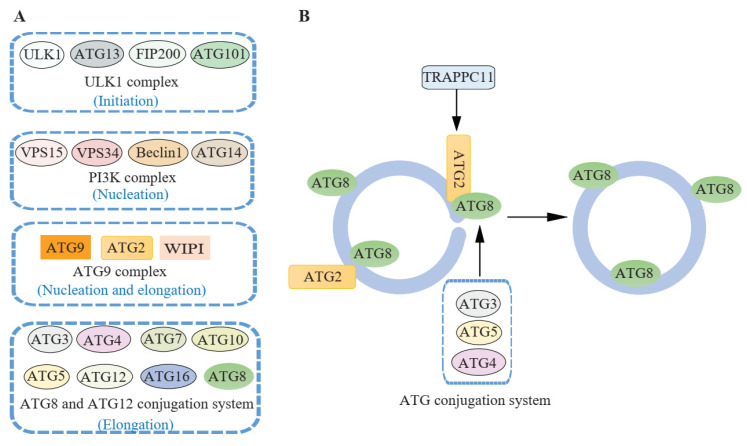
ATG proteins involved in autophagosome closure. (**A**) The ULK1 complex is required for the initiation of autophagy. The PI3K complex is responsible for PI3P production and essential for nucleation of the IM. The ATG9 complex consisting of transmembrane protein ATG9, lipid transfer protein ATG2 and PI3P-effector ATG18 (WIPIs) are required for both the nucleation and elongation steps. Two ubiquitin-like conjugation systems (ATG8 proteins conjugation to PE and the conjugation of ATG12–ATG5) play important roles in the elongation step. Mammalian ATG proteins including LC3 and the GABARAP subfamilies are shown as ATG8 for simplicity. (**B**) The ATG conjugation system including ATG3, ATG4, ATG5, ATG8 (GABARAPs) and the lipid transfer protein ATG2 are involved in autophagosome closure. In the ATG8 conjugation system, ATG3 acts as an E2-like enzyme. The ATG12–ATG5–ATG16 complex acts as an E3-like enzyme for the conjugation of ATG8 proteins (LC3 and GABARAP subfamilies) to PE. ATG4 cleaves ATG8 proteins to expose a glycine residue before conjugation, and cleaves ATG8–PE for the recycling of ATG8 proteins. The interaction between ATG2 and GABARAPs is also critical for the closure step. TRAPPC11, a TRAPP III-specific subunit, is required for the recruitment of ATG2 to isolation membranes.

Autophagy-related (ATG) proteins constitute the core machinery for autophagy. The ULK1/Atg1 complex is required for the initiation of autophagy. The PI3K complex is responsible for PI3P production and essential for nucleation of the IM. The ATG9 complex consisting of transmembrane protein ATG9, lipid transfer protein ATG2, and PI3P-effector ATG18 (WIPIs) is required for both the nucleation and elongation steps. Two ubiquitin-like conjugation systems (ATG8 proteins conjugated to PE and the conjugation of ATG12–ATG5) play important roles in the elongation step. In the ATG12–ATG5 conjugation system, ATG7 and ATG10 function as E1- and E2-like enzymes, respectively. In the ATG8 conjugation system, ATG7 functions as an E1-like enzyme, and ATG3 acts as an E2-like enzyme. The ATG12–ATG5–ATG16 complex acts as an E3-like enzyme for the conjugation of ATG8 proteins (LC3 and GABARAP subfamilies) to PE. ATG8 proteins are cleaved by ATG4 to expose a glycine residue before conjugation, and ATG8–PE is also cleaved by ATG4, which allows the recycling of ATG8 proteins [1,2,3,4,5,6]. Several ATG proteins were reported to be involved in autophagosome closure. The accumulation of unclosed isolation membranes has been observed in in ATG3-deficient MEFs [21], ATG5-deficient MEFs and HeLa cells [24,25], ATG2-deficient HeLa cells [27], NIH-3T3 cells overexpressing a dominant negative mutant Atg4B^C74A^ [22], HeLa cells overexpressing a dominant negative mutant Atg4A^C77A^ [23], and U-2 OS cells expressing an ATG2A-mLIR mutant, which blocks ATG2-GABARAP interaction [26]. These findings suggest that the ATG conjugation system and the lipid transfer protein ATG2 are essential for the closure step (Figure 3, Table 1).

### 3.1. ATG3

Atg3 is an E2-like enzyme involved in the conjugation of Atg8 homologues, including microtubule-associated protein 1 light chain 3 (LC3), GABA receptor-associated protein (GABARAP), and Golgi-associated ATPase enhancer of 16Kd (GATE-16) with phosphatidylethanolamine (PE). Atg3 deficiency blocks the recruitment of LC3 to IMs and leads to the accumulation of unclosed IM structures in Atg3-deficient mouse embryonic fibroblasts (MEFs) [21]. The average size of the autophagic structures in ATG3-deficient MEFs is smaller than that in wild-type MEFs cultured under starvation conditions. The induction of autophagosome-like structures in mutant MEFs is suppressed by pretreatment with wortmannin, suggesting that these autophagosome-like structures are also derived from autophagic mechanisms involving the function of phosphatidylinositol 3-kinases. 

### 3.2. ATG4

The cysteine protease Atg4 cleaves the C terminal of Atg8 to expose a glycine residue before conjugation to PE. Atg8–PE is also cleaved by Atg4, which allows the recycling of Atg8 proteins [36]. In 2008, Fujita et al. reported that the overexpression of a dominant negative mutant Atg4B^C74A^ inhibits LC3–PE conjugation and results in the accumulation of unclosed IMs, which are labelled with Atg5 [22]. The length of these structures is comparable with the length of autophagosomal membrane structures in control cells. Atg4B^C74A^ overexpression affects PE conjugation of all Atg8 family members, as Atg4B recognizes all mammalian Atg8s [37]. In 2010, Weidberg et al. used a dominant negative mutant of Atg4A (Atg4A^C77A^), a protease specific to GABARAPs, and found that overexpression of Atg4A^C77A^ leads to an increase in the number of open autophagic membranes labelled with Atg16 [23]. 

### 3.3. ATG5

The Atg12–Atg5 conjugate acts as an E3-like enzyme for the conjugation of Atg8s to PE and forms a complex with Atg16 [38]. In ATG5-deficient MEFs and HeLa cells, isolation membranes can elongate and bend while they stay associated with the ER, and cannot seal to form autophagosomes. The morphology of isolation membranes in ATG5-deficient MEF is almost normal. In contrast, isolation-membrane-like structures are not detected in ATG9A-knockout cells, FIP200-knockout cells, or cells treated with wortmannin, which inhibits PtdIns 3-kinase activity. These data suggest that the closure step requires ATG5, whereas FIP200, ATG9A, and PtdIns 3-kinase are essential for IM formation [24].

### 3.4. GABARAPs

Mammalian Atg8s can be categorized into the LC3 (LC3A-C) and GABARAP/GATE-16 (GABARAP, GABARAPL1, GABARAPL2/GATE-16) subfamilies. Although both subfamilies are essential for autophagy, LC3s and GABARAPs do not complement each other and act differently during autophagosome biogenesis [23]. Knockdown and overexpression of the LC3 and GABARAP subfamilies yields opposite effects on the size and number of IMs. The overexpression of a dominant negative mutant of Atg4A (Atg4A^C77A^), a protease specific for GABARAPs, results in the accumulation of larger open autophagic membranes, suggesting that GABARAPs act in autophagosome closure without affecting IM elongation [23].

### 3.5. ATG2

In the budding yeast *Saccharomyces cerevisiae*, prApe1, the cargo of Cvt vesicles and the autophagosome, accumulates in the atg2Δ strain. prApe1 is protease-accessible, not protected by the Cvt vesicle or autophagosome, indicating Atg2 is required for the expansion or closure of the Cvt vesicle or autophagosome [28]. In HeLa cells lacking both ATG2A and ATG2B, Velikkakath et al. observed the accumulation of unclosed autophagosome-related membranes that colocalize with Atg9A, ULK1, Atg14, WIPI1, Atg5, and LC3. GFP-LC3-II and p62 are also sensitive to proteinase K, suggesting that autophagosome closure is impaired [27]. In 2020, Bozic et al. reported that the interaction between ATG2 and GABARAPs is critical for IM closure. They identified a highly conserved LC3 interaction region (LIR) in ATG2A and ATG2B, which mediates the interaction between human ATG2 and GABARAP proteins. Using the proteinase K protection assay and syntaxin 17 (STX17) translocation assay, they showed that the ATG2A–GABARAP interaction mutants were unable to form and close IMs. The ATG2A–LIR mutant leads to the accumulation of immature and open IMs, whereas ATG2A–WIPI4 interaction is dispensable for autophagosome formation and autophagy flux [26].

## 4. Rab GTPases and SNAREs

Rab GTPases are key regulators of membrane traffic in eukaryotic cells [39,40]. They are molecular switches that cycle between an inactive (GDP-bound) and active form (GTP-bound). Guanine nucleotide exchange factors (GEFs) control this switch by stimulating the release of GDP from the Rab and accelerating the uptake of GTP. Once the Rab GTPase is membrane-bound and active, it can interact with effectors including tethers, cytoskeletal motors, and soluble N-ethylmaleimide sensitive factor attachment protein receptors (SNAREs). SNAREs constitute the core machinery for membrane fusion [41]. Depending on whether the SNARE motif contains a conserved glutamate (Q) or an arginine (R) residue, SNAREs are classified into Q-SNAREs or R-SNAREs. SNAREs localize on opposing membrane compartments, and membrane fusion is driven by the assembly of a trans-SNARE complex consisting of one R-SNARE and three Q-SNAREs (Qa, Qb, Qc) [41,42,43]. Rab GTPases and SNAREs are implicated in multiple steps in autophagy [44,45,46]. Several recent studies implied a role for Rabs and SNAREs in the closure of the IM to form a sealed autophagosome.

### 4.1. Vps21 (RAB5) GTPase Module

The ESCRT-III subunit Snf7 (CHMP4 in mammals) interacts with Atg17 (FIP200 in mammals) and leads to the recruitment of ESCRT to IMs. This process is essential for autophagosome closure, and it is dependent on Rab GTPase Vps21 (RAB5) [19,20]. The endocytic Vps21 GTPase module including the Rab GTPase Vps21, the GEF Vps9, the CORVET tethering complex subunit Vps8, and SNARE protein Pep12 is required for autophagosome closure in budding yeast [29]. The deletion of these genes results in the accumulation of unsealed autophagosomes without affecting the formation of Atg8–PE and IM elongation [29].

### 4.2. Syntaxin 13

Lu et al. identified the SNARE syntaxin13 as a strong genetic modifier of toxicity caused by mutant CHMP2B, an ESCRT-III component that causes frontotemporal dementia and accumulation of autophagic membrane structures. Syntaxin13 strongly enhanced the CHMP2B^Intron5^ eye phenotype in *Drosophila melanogaster* [30]. Syntaxin13 is a BLOC-1-interacting recycling endosomal SNARE [47,48] that was identified as a regulator of autophagosome closure in HEK293 and HeLa cells. Syntaxin13 is present on IMs, and its knockdown leads to the accumulation of LC3 and Atg5-positive puncta. Results of the mCherry-GFP-LC3 assay indicate that the defects caused by the loss of Syntaxin13 occur before the fusion with lysosomes. Syntaxin13- and LC3-positive multilamellar structures accumulate in cells with dysfunctional ESCRT-III, indicating that Syntaxin13 functions upstream of ESCRT-III in autophagosome closure. Although Syntaxin13 is a strong genetic modifier of mutant CHMP2B, there is no direct physical interaction between these proteins in *Drosophila melanogaster* or HEK293 cells [30].

### 4.3. TRAPC11

Transport protein particle (TRAPP) complexes are multi-subunit tethering complexes conserved in eukaryotes that act as GEFs (GTP exchange factors) for RAB1. Yeast TRAPP complexes are well characterized, with three complexes known to play different roles in membrane trafficking. TRAPPI mediates ER–Golgi transport, and TRAPPII regulates endosome–Golgi transport. TRAPPIII, via its Trs85 subunit, has been linked to autophagy [49]. In mammalian cells, there are two TRAPP complexes related to yeast; TRAPPII and TRAPPIII. All yeast subunits are conserved in higher eukaryotes; however, metazoans contain several subunits not found in *Saccharomyces cerevisiae*, including TRAPPC11 and TRAPPC12, both of which are TRAPP III-specific subunits [50]. Stanga et al. reported that TRAPPC11 interacts with ATG2B–WIPI4 in an ATG9A-dependent manner. A portion of TRAPPC11 localizes to isolation membranes and recruits ATG2B–WIPI4 to preautophagosomal membranes. TRAPPC11 depletion results in the accumulation of unsealed isolation membranes, a phenotype similar to that of ATG2-depleted cells [31]. In contrast, TRAPPC12 is active after autophagosome formation, and the depletion of TRAPPC8 affects the formation of isolation membranes, which is consistent with a previous report showing that depletion of TRAPPC8 acts early in autophagosome formation and disrupts ATG9 trafficking [31,51].

### 4.4. CK1δ/Hrr25 Kinase

CK1δ (casein kinase I δ), a member of the CK1 family of serine/threonine specific kinases, is involved in the regulation of various cellular processes including circadian rhythms, Wnt signaling, cytoskeleton maintenance, the cell cycle, and DNA damage repair [52]. Hrr25, the yeast homologue of CK1δ, has been reported to activate multiple selective autophagy pathways by phosphorylating cargo receptors and promoting the interactions of these receptors with the scaffold protein Atg11 [53,54,55]. We previously reported that CK1δ/Hrr25 is also required for macroautophagy as an effector of Rab1/Ypt1 [56]. CK1δ depletion results in the accumulation of unclosed isolation membranes and increased association of LC3 with ATG9A, ATG14L, and ATG16L1 in HeLa cells. In budding yeast, elongated and unclosed isolation membranes accumulate in the temperature-sensitive mutant hrr25-5 under starvation conditions, indicating that CK1δ/Hrr25 kinase is involved in autophagosome closure [32].

## 5. Sphingomyelin

Sphingomyelin is an essential cellular lipid that traffics between the plasma membrane, Res, and TGN. It is degraded to ceramide and phosphocholine by sphingomyelin phosphodiesterase 1 (SMPD1). Mutations in the SMPD1 gene result in Niemann–Pick disease type A and B, characterized by sphingomyelin accumulation and disturbed tissue homeostasis. The accumulation of elongated and unclosed autophagic membrane structures was observed in Niemann–Pick type A patient fibroblasts [33]. The treatment of healthy control cells with exogenous sphingomyelin as well as the depletion of SMPD1 in MCF7 breast cancer cells also leads to the accumulation of unclosed autophagic membranes, which are WIPI2-, ATG16L1-, and LC3B-positive. ATG9A trafficking from the recycling endosome is also disturbed in SMPD1-deficient cells and sphingomyelin-treated cells [33].

## 6. Calcium

Calcium acts as a ubiquitous intracellular messenger that influences various aspects of cellular life [57]. Although it is well established that calcium is an important regulator of autophagy, both stimulatory and inhibitory functions for Ca^2+^ towards autophagy have been proposed [58,59,60]. Elevated cytosolic Ca^2+^ concentrations lead to autophagy stimulation through multiple pathways involving calmodulin-dependent kinase kinase β (CaMKKβ), AMPK, mTOR, inositol 1,4,5-trisphosphate receptor (IP3R), protein kinase C theta (PRKCQ), and other proteins [58,59,60]. Cytosolic Ca^2+^ chelation with BAPTA-AM strongly prevents the induction of autophagy, suggesting that Ca^2+^ plays an important role in the induction or activation of autophagy. Conversely, there are a number of studies reporting that Ca^2+^ suppresses autophagy through pathways involving IP3R, Beclin1, AMPK, mTOR [58,59,60], which makes the role of calcium in autophagy complicated and controversial, possibly depending on spatiotemporal and amplitude characteristics of calcium signals as well as the states of cells.

In 2013, Engedal et al. explored the relationship between cytosolic calcium concentration and autophagy, using two agents that increase intracellular calcium concentration, namely the calcium ionophore A23187 that allows calcium ions to cross biological membranes and the sarco/endoplasmic reticulum Ca^2+^-ATPase (SERCA) inhibitor thapsigargin, which blocks calcium transport from the cytosol into the ER. They found that these two calcium modulators induce a block in autophagic flux after WIPI1 recruitment, but before the autophagosome closure, which leads to the production of unclosed autophagic structures [34]. They also found that the inhibitory effect of A23187 and thapsigargin is independent of ER stress or bulk changes in cytosolic calcium levels, and proposed that an increased intracellular calcium level blocks autophagosome formation at the stage of expansion and closure. Zhao et al. reported that SERCA is regulated by the ER-localized transmembrane protein VMP1 and controls ER–IM contact for autophagosome formation as well as ER contact with lipid droplets, mitochondria, and endosomes. The loss of VMP1 or thapsigargin treatment causes the stable association of IMs with the ER, which blocks the detachment of IM from the ER and autophagosome completion [61]. Thus, although the treatment of calcium modulators A23187 and thapsigargin leads to a phenotype that is very similar to loss of regulators of autophagosome closure, the effect might result from the dysregulation of ER–IM contacts instead of the closure defect.

## 7. Concluding Remarks

Autophagosome closure is one of the least well-characterized events in autophagy. One reason is that it is not easy to distinguish fully closed autophagosomes from unclosed isolation membranes due to technical limitations. Moreover, some regulators of autophagosome closure are also involved in the elongation step, which makes it possible that the closure defect is secondary to the elongation defect. Different techniques including electron microscopy, super-resolution microscopy, the protease protection assay, the HaloTag-LC3-based autophagosome completion assay, the optogenetic closure assay have been used to distinguish closed autophagosomes from unclosed isolation membranes and identify regulators of autophagosome closure. However, there is no assay that can directly separate elongation defects and closure defects if the candidate protein is involved in both steps.

Recent progress in the field has elucidated that the closure of autophagosomes is tightly regulated by ESCRT complexes, ATG proteins, SNAREs, Rab GTPase, Rab-related proteins, sphingomyelin, and calcium. However, a detailed molecular mechanism regarding the closure process remains elusive, and several fundamental issues remain to be addressed. Although the role of the ESCRT complex in autophagosome closure has been shown from yeasts to humans, and several upstream factors including Vps21, Syntaxin13 and Atg17 have been identified, how ESCRT machinery is specifically recruited to the edge of isolation membranes and how this process is regulated remains unclear. The rim of the IM must be sufficiently narrow to be cleaved by ESCRT-III. Which proteins trigger the ESCRT machinery to initiate scission and regulate the biochemical activity of ESCRT during autophagosome closure remains a question to be addressed. Multiple components of ATG conjugation system play roles in the autophagosome closure, but some ATG proteins like ATG3 are also required for the elongation step. Hence, it is unclear if the accumulation of the unclosed IMs in Atg3-deficient cells results from elongation defects. The molecular mechanism of ATG2 and GABARAP-regulated scission is poorly understood, although ATG2–GABARAP interaction and formation of GABARAP–PE clearly play a role in autophagosome closure. We still do not understand why different subunits of the mammalian TRAPPIII complex (TRAPPC8, TRAPPC11, and TRAPPC12) act at different sites in the autophagy pathway and how TRAPPC11 specifically regulates the closure step. The substrates that CK1δ/Hrr25 phosphorylates during autophagosome closure remain unknown, as none of the reported substrates of CK1δ/Hrr25 kinase have a phenotype in autophagosome closure. The mechanism of sphingomyelin and calcium-regulated closure remains elusive. Furthermore, we cannot exclude the possibility that the closure defect in thapsigargin-treated cells results from dysregulated ER–IM contacts.

To obtain a complete picture of autophagosome closure, further regulators of autophagosome closure must be identified. High-throughput screening and identification of novel binding partners of reported regulators could provide more information about the regulators of autophagosome closure. To characterize the regulators of autophagosome closure, it is important to check whether the IM is properly elongated. If loss of the candidate protein leads to blocking autophagosome closure without any elongation defect, the candidate protein clearly functions in the closure step. If the candidate protein is required for both the elongation and closure steps, the accumulation of unclosed IMs could be secondary to the elongation defect. Although there is no assay that can directly separate the elongation defect and closure defect if the candidate protein is involved in both steps, there are some methods which can provide clues about whether the closure defect results from the elongation step. The systematic analysis of mutations or truncations of candidate proteins for both closure and elongation defects may identify some specific mutations or truncations that are required for closure but dispensable for elongation or vice versa. An interaction with other regulators of IM closure may be an indication that candidate protein also participates in the closure step. The analysis of biological function and development of new techniques will also help to distinguish the elongation and closure defects. For example, the HaloTag-LC3-based autophagosome completion assay has established a direct role for the ESCRT machinery in autophagosome closure, that was consistent with its role in membrane scission [16,17].

Key questions also remain regarding the mechanisms by which the regulators of closure collaborate with each other and their spatial and temporal regulation. The characterization of the hierarchy of regulators of closure, functional assessment of interactions between them, and establishment of in vitro reconstitution systems will provide the means to determine the full picture related to autophagosome closure. Furthermore, as the IM must elongate to the proper size before closure, and as the fusion step must be prevented until closure completes, an essential question concerns how closure is correctly synchronized with the expansion and fusion steps. It is also important to understand how the mechanism of autophagosome closure varies between different types of autophagy and different physiological and pathological conditions. The elucidation of these aspects will further advance the understanding of the mechanisms involved in autophagosome closure.

## Figures and Tables

**Table 1:** Key regulators of autophagosome closure.

Protein	Orgamism and Cell Type	Phenotype
**ESCRT machinery**		
CHMP2A (ESCRT-III subunit)	U-2 OS	Depletion of CHMP2A leads to the accumulation of unclosed IMs in macroautophagy and mitophagy, and the accumulation of CHMP4 on mitophagosomes [16,18].
VPS4 (ATPase)	U-2 OS	The expression of a dominant negative mutant of VPS4A (VPS4A^E228Q^) results in the accumulation of unclosed IMs [16].
VPS37A (ESCRT-I subunit)	U-2 OS	VPS37A translocates to the IM and recruits the ESCRT-I subunit VPS28 and the ESCRT-III subunit CHMP2A to it. Loss of VPS37A leads to the accumulation of unclosed IMs [17].
CHMP4B (ESCRT-III subunit)	RPE-1	CHMP4B is recruited to the unsealed autophagosome during macroautophagy and mitophagy and accumulates on mitophagosomes in CHMP2A-deficient cells [18].
Vps4 (ATPase)	*Saccharomyces cerevisiae*	Depletion results in the accumulation of unsealed IMs decorated with multiple Atg proteins [19,20].
Snf7 (ESCRT-III subunit)	*Saccharomyces cerevisiae*	Snf7 interacts with Atg17 or Atg11 and leads to ESCRT recruitment to IM. Loss of Snf7 results in the accumulation of unsealed IMs [19,20].
**ATG proteins**		
ATG3	MEF	ATG3 deficiency blocks the recruitment of LC3 to IMs, impairs the elongation and closure of IMs and leads to the accumulation of unclosed IM structures [21].
ATG4A, B	NIH-3T3, HeLa	Overexpression of a dominant negative mutant Atg4B^C74A^ inhibits LC3–PE conjugation and results in the accumulation of unclosed IMs [22]. Overexpression of Atg4A^C77A^ leads to an increase in the number of open IMs labelled with Atg16 [23].
ATG5	MEF, HeLa	IMs can elongate and bend but can’t seal to form autophagosomes in ATG5-deficient cells [24,25].
GABARAPs	HeLa, U-2 OS	In GABARAP-depleted cells, autophagosome biogenesis is impaired and the Atg5-labelled structures are significantly larger. Overexpression of a dominant negative mutant Atg4A^C77A^, a protease specific for GABARAPs, results in the accumulation of open IMs [23]. ATG2A-mLIR mutant, which blocks ATG2-GABARAP interaction leads to the accumulation of open IMs [26].
ATG2	HeLa, U-2 OS	Unclosed IMs accumulate in HeLa cells lacking both ATG2A and ATG2B [27]. The expression of ATG2A-LIR mutant which blocks the ATG2A-GABARAP interaction leads to the accumulation of open IMs [26].
Atg2	*Saccharomyces cerevisiae*	The expansion or closure of the Cvt vesicle or autophagosome is impaired in the atg2Δ strain [28].
**Rab GTPases and SNAREs**		
Vps21 module (Rab GTPase Vps21, the GEF Vps9, the CORVET subunit Vps8, and SNARE protein Pep12)	*Saccharomyces cerevisiae*	The deletion of these genes results in the accumulation of unsealed autophagosomes without affecting the formation of Atg8–PE and IM elongation [29].
Syntaxin 13 (SNARE)	*Drosophila melanogaster,* HEK293, HeLa	Syntaxin 13 is a strong genetic modifier of CHMP2B in *Drosophila melanogaster* and functions upstream of ESCRT-III in autophagosome closure. Knockdown of syntaxin 13 leads to the accumulation of IMs in HEK293 and HeLa cells. There is no direct physical interaction between Syntaxin 13 and CHMP2B in *Drosophila melanogaster* or HEK293 cells [30].
TRAPC11 (TRAPP III subunit)	HeLa	A portion of TRAPPC11 localizes to IMs and recruits ATG2B-WIPI4. TRAPPC11 depletion results in the accumulation of unsealed isolation membranes [31].
CK1δ/Hrr25 kinase (Rab1/Ypt1 effector)	HeLa, *Saccharomyces cerevisiae*	CK1δ depletion results in the accumulation of unclosed isolation membranes in HeLa cells. In budding yeast, elongated and unclosed IMs accumulates in the temperature-sensitive mutant *hrr25-5* under starvation conditions [32].
**Others**		
Sphingomyelin	Niemann-Pick type A patient fibroblast, MCF7	The accumulation of elongated and unclosed autophagic membrane structures was observed in Niemann-Pick type A patient fibroblasts, healthy control cells treated with exogenous sphingomyelin, SMPD1-depleted MCF7 breast cancer cells [33].
Calcium	LNCaP, U-2 OS	The calcium ionophore A23187 or SERCA inhibitor thapsigargin treatment induces a block in autophagic flux before the autophagosome closure and leads to the production of unclosed IMs [34].

Notes: IM, isolation membrane; PE, phosphatidylethanolamine; SMPD1, ceramide and phosphocholine by sphingomyelin phosphodiesterase 1; SERCA, the sarco/endoplasmic reticulum Ca^2+^-ATPase; U-2 OS, human osteosarcoma cells; RPE-1, retinal pigment epithelial cells.

## Abbreviations

IM: isolation membrane; PE, phosphatidylethanolamine; SMPD1, ceramide and phosphocholine by sphingomyelin phosphodiesterase 1; SERCA, the sarco/endoplasmic reticulum Ca^2+^-ATPase.
